# Factors Affecting the Intention to Modify Lifestyle in the Cardiovascular Disease Risk Group in Korea

**DOI:** 10.3390/healthcare9050496

**Published:** 2021-04-22

**Authors:** JaeLan Shim, KyungAe Kim

**Affiliations:** 1 Department of Nursing, Dongguk University, Seoul 38066, Korea; jrshim@dongguk.ac.kr; 2 College of Nursing, Kyungdong University, Wonju 26495, Korea

**Keywords:** cardiovascular disease, FRS, lifestyle modification, risk, intention

## Abstract

This study aimed to identify people at risk of cardiovascular diseases (CVD) using the Framingham risk score (FRS) and to examine their willingness to modify their lifestyle. A descriptive correlational study with 1229 participants, aged 30–74 years, without cardiovascular or cerebrovascular diseases who visited a health examination center in South Korea was conducted. Of 1229 participants, 455 were identified as high risk for CVD with an FRS of 10 or higher. A logistic regression analysis confirmed age, sex, muscle training, and weekly drinking frequency as predictors of intention to quit smoking; age, smoking, and waist to height ratio (WHtR) as predictors of intention to quit drinking; sex and WHtR as predictors of intention to engage in physical exercise; and hypertension and body mass index as predictors of intention of muscle training. People at high risk for CVD lack willingness to modify their lifestyle, and aggressive, customized intervention is needed to promote lifestyle modification.

## 1. Introduction

Cardiovascular disease (CVD) is the leading cause of death worldwide. According to the 2019 Statistics Korea report, it is the second leading cause of death following malignant neoplasm, with 117.4 deaths per 100,000 people [1]. Chronic diseases such as CVD are multifactorial and have multiple etiologies, rendering early diagnosis difficult. Furthermore, the time of disease onset is unclear and there is a long latent period. Thus, prevention, as opposed to treatment, is the focus for chronic diseases, which calls for screening of people at a high risk for CVD [2].

In 2014, Korea designated the first week of every September as the cardiovascular and cerebrovascular diseases prevention week, during which the government strives to reduce CVD mortality by launching the Red Circle Campaign. It increases awareness of and promotes compliance with the prevention and management of severe cardiovascular diseases such as myocardial infarction and stroke [3].

Considering that heart disease remains one of the leading causes of death despite advances in medical technology and development of novel drugs, it is important to note that other factors contribute to the incidence of heart disease. [4]

While many countries have strived to regulate these CVD risk factors and have announced their success in reducing CVD mortality [4], the Korean National Health and Nutrition Examination Survey (KNHANES) reports that health management pertaining to diseases that contribute to CVD morbidity, such as diabetes mellitus (DM), hypertension, and hyperlipidemia, is inadequate in Korea [5]. This highlights the need for more aggressive and preventive efforts in the country.

Cardiovascular disease risk factors can be categorized into two groups: modifiable and non-modifiable risk factors. Non-modifiable cardiovascular disease risk factors are those that cannot be changed. These include a person’s age, ethnicity, and family history (genetics cannot be changed), among other factors. Modifiable cardiovascular disease risk factors are those that can be reduced or controlled with behavioral changes [6].

Some of the preventable risk factors of CVD include smoking, hypertension, DM, hyperlipidemia, obesity, and lack of exercise [7,8,9]. Furthermore, people who abuse alcohol are at 1.5–2.0-times greater risk of developing an ischemic heart disease compared to their non-drinking counterparts [7,10]. Therefore, the importance of CVD-related lifestyle modification has been emphasized.

The Framingham risk score (FRS) is a simple tool widely used to predict the level of CVD risk in the next 10 years, and it provides guidelines for managing risk factors [11,12]. In particular, the FRS for Coronary Heart Disease (FRS-CHD) predicts the incidence of coronary heart disease, including death from angina pectoris, myocardial infarction, and coronary artery disease, in the next 10 years, by sex in adults aged 30–74 years [13]. This tool accurately and efficiently predicts CVD and the risk of death because it assesses multiple factors, such as age, smoking, DM, systolic (SBP) and diastolic blood pressure (DBP), total cholesterol, and high-density lipoprotein cholesterol (HDL-C) [12,13].

A Korean study using FRS confirmed the factors affecting FRS by sex [14], and another study identified the factors influencing middle-aged women’s lifestyle habits according to their FRS level that affect their 10-year cardiovascular disease risk [15]. Other studies have explored the relationship between FRS and carotid intima media thickness [16] and the relationship between the Fracture Risk Assessment Tool and FRS [17]. The findings of these studies suggest the need for a large-scale prospective study.

The purpose of the present study was to classify risk groups using FRS and to identify the factors that influence the willingness to improve lifestyle, which is a modifiable risk factor. We hypothesized the following:

- Among participants with moderate or high cardiovascular risk, the better the lifestyle habits, the higher their intention to quit smoking.
- Among participants with moderate or high cardiovascular risk, the better the lifestyle habits, the higher their intention to stop drinking.
- Among participants with moderate or high cardiovascular risk, the better the lifestyle habits, the higher their intention to engage in physical activity.
- Among participants with moderate or high cardiovascular risk, the better the lifestyle habits, the higher their intention to engage in strength exercises.

## 2. Materials and Methods

### 2.1. Research Design

This study employed a cross-sectional study design to identify, through influencing with CVD risk, participants’ intentions to modify their lifestyle habits.

### 2.2. Setting and Sample

**Figure 1:**
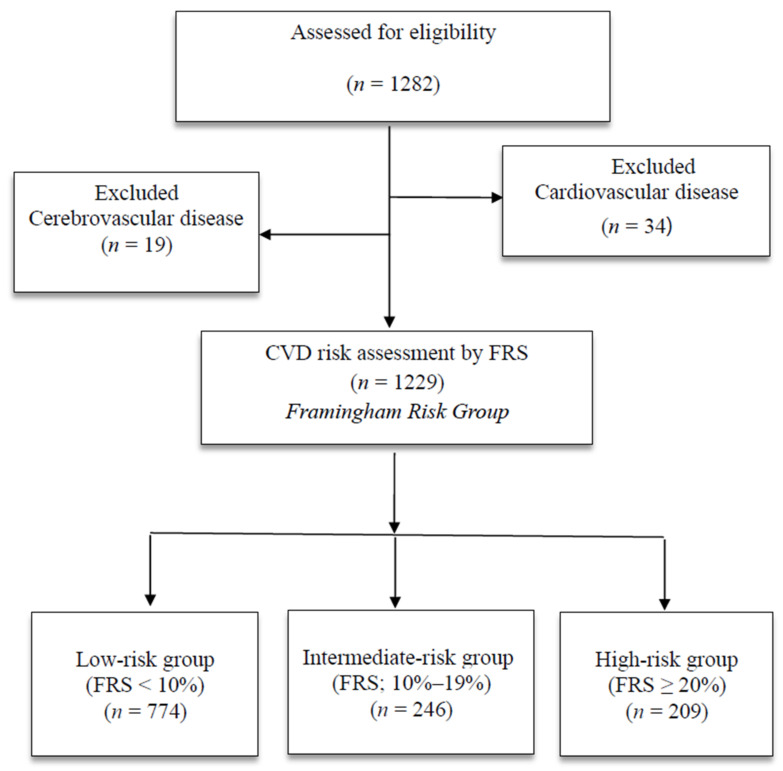
Flow chart of the study population; HDL = high-density lipoprotein.

A total of 1282 people aged 30–74 years [13] who underwent a health examination at a health examination center in Korea from June 1 to July 31, 2020 were enrolled in this study. Data were collected via physical examination, blood tests, and a self-report questionnaire. A total of 34 individuals diagnosed with a heart disease (e.g., angina, myocardial infarction) or currently taking medications and 19 people diagnosed with and currently taking medications for cerebrovascular disease (stroke, cerebral hemorrhage) were excluded from the study [13]. As a result, a total of 1229 participants were included in the analysis (Figure 1).

### 2.3. Measurements

#### 2.3.1. General and Lifestyle-Related Characteristics

Participants’ demographic and lifestyle-related information, namely, age, sex, drinking, and smoking, was surveyed using the pre-exam health questionnaire. Information about physical activity was collected using the International Physical Activity Questionnaire (IPAQ) [18], specifically the number of times and duration of high-intensity physical activity that caused shortness of breath in the past week (e.g., running, aerobics, cycling, carrying things using the stairs) and moderate-intensity physical activity that caused mild shortness of breath (e.g., fast walking, tennis doubles, carrying light objects, cleaning). In accordance with the American Heart Association (AHA) Guidelines [19], people who engage in at least 150 min of moderate-intensity physical activity per week, 75 min of high-intensity physical activity per week, or at least two sessions of muscle training per week are considered to be engaging in sufficient physical activity.

#### 2.3.2. Hematologic Test

For the blood test, the participants were informed in advance to refrain from overeating, drinking, and smoking, and to skip their medications as they may affect the test results. All participants fasted for at least eight hours prior to the blood test. Fasting glucose, HDL-C, total cholesterol, aspartate aminotransferase (AST), gamma glutamyl transpeptidase (GGT), and creatinine (Cr) levels were analyzed. Blood tests were performed by three clinical pathologists with more than 10 years of experience working at the institution where this study was conducted. The pathologists receive a training session once a year on drawing blood accurately.

#### 2.3.3. Intention to Improve Lifestyle

Participants’ intentions to improve their lifestyle pertaining to smoking, drinking, physical exercise, and muscle training were examined using a self-report questionnaire, with “yes” or “no” responses.

#### 2.3.4. Framingham Risk Score-Coronary Heart Disease (FRS-CHD)

The FRS was calculated by scoring participants’ age, HDL-C, total cholesterol, SBP, DM, and smoking status according to their sex, as per the calculation method presented in the Framingham heart study [13]. This calculation method classifies people into 10 age groups in 4-year units from the age of 30. In step 2, people are classified into five groups according to total cholesterol and HDL-C by sex. In step 3, people are divided into six groups according to SBP by sex. In step 4, people are scored according to DM and smoking status (non-smoker, current smoker). In step 5, the scores are summed to calculate the total score, based on which an individual’s 10-year risk for cardiovascular and cerebrovascular disease is estimated. The risk estimate is then used to classify the participants into low-risk (<FRS 10%), intermediate-risk (10–19%), and high-risk groups (≥20%) [12].

### 2.4. Ethical Considerations

This study was approved by the Institutional Review Board of the research facility (IRB No. 130750-202002-HR-001) prior to data collection. After explaining the purpose of this study to the research participants, written informed consent was obtained from the participants prior to the health examination.

### 2.5. Statistical Analysis

The data were analyzed using SPSS/WIN 23.0 software (IBM SPSS Statistics, Chicago, IL, USA). The general characteristics were analyzed using descriptive statistics, namely, frequency and percentage. Differences in demographics and willingness to modify lifestyle according to the CVD risk category were analyzed with a one-way analysis of variance (ANOVA). The predictors of willingness to modify lifestyle in 455 participants in the intermediate-risk and high-risk groups (FRS ≥ 10%) were analyzed using binary logistic analysis.

## 3. Results

### 3.1. Differences in General Characteristics and Intention to Modify Lifestyle According to CVD Risk Category

The mean age significantly differed among the low-risk group (54.6 ± 10.7 years), intermediate-risk group (62.0 ± 7.7 years), and high-risk group (65.4 ± 5.7 years) (*F* = 135.1, *p* < 0.001). In terms of sex, 667 (87.5%) in the low-risk group and 149 (60.6%) in the intermediate-risk group were women, while 184 (88.0%) in the high-risk group were men.

The prevalence of DM significantly differed among the low-risk group (n = 34, 4.4%), intermediate-risk group (*n* = 42, 20.6%), and high-risk group (*n* = 47, 22.5%) (*F* = 76.87, *p* < 0.001). The percentage of current smokers also significantly differed in the high-risk group (*n* = 158, 75.6%) compared to the low-risk and intermediate-risk groups (*F* = 380.34, *p* < 0.001). The percentage of people who drank alcohol at least once a week was significantly higher in the high-risk group (*n* = 87, 41.6%) (*F* = 43.88, *p* < 0.001). In terms of intention to quit smoking, the majority in all three groups had no intention to quit smoking, with a significant difference among the groups (*F* = 56.55, *p* < 0.001). In terms of intention to quit drinking, only 28.2% of the high-risk group were willing to quit drinking, with a significant difference among the three groups (*F* = 8.78, *p =* 0.012) (Table 1).

### 3.2. Differences in Blood Test Results According to FRS Category

Table 2 shows the differences in the blood test results according to the FRS category. Body mass index (BMI) was highest in the high-risk group (24.9 ± 2.9 kg/m^2^), with a significant difference among the three groups (F = 16.61, *p* < 0.001). Waist to height ratio (WHtR) was high in the intermediate- and high-risk groups (0.53 ± 0.1), with a significant difference among the three groups (F = 15.94, *p* < 0.001).

### 3.3. Predictors of Intention to Modify Lifestyle in At-Risk CVD Groups

The predictors of intention to modify lifestyle in at-risk CVD groups were analyzed for 455 participants in the intermediate-risk (FRS ≥ 10%) and high-risk groups (FRS ≥ 19%) (Table 3).

### 3.3.1. Predictors of Intention to Quit Smoking in At-Risk CVD Groups

The model for identifying the predictors of intention to quit smoking in the at-risk CVD groups was significant (χ^2^ = 294.09, *p* < 0.001), with 41.8% of the variance explained (Table 3). Intention to quit smoking was 1.15-times higher with older age (OR: 1.15, 95% CI: 1.11–1.20) and 5.95-times higher among women than men (OR: 5.95, 95% CI: 3.18–11.13). Intention to quit smoking was 1.17-times higher among those who engaged in more muscle training (OR: 5.95, 95% CI: 1.03–1.33) and 1.27-times higher among those with a higher weekly drinking frequency (OR: 1.15, 95% CI: 1.11–1.20) (Table 3).

### 3.3.2. Predictors of Intention to Quit Drinking in At-Risk CVD Groups

The model for identifying the predictors of intention to quit drinking in the at-risk CVD groups was significant (χ^2^ = 15.84, *p* < 0.001), with 24.3% of the variance explained (Table 3). Intention to quit drinking was 1.08-times higher with older age (OR: 1.08, 95% CI: 1.05–1.10) and 3.78-times higher among smokers than non-smokers (OR: 3.78, 95% CI: 2.39–6.00). Intention to quit drinking was 0.68-times lower among those with higher WHtR than those with lower WHtR (OR: 0.68, 95% CI: 0.48–0.96) (Table 3).

### 3.3.3. Predictors of Intention to Engage in Physical Exercise and Muscle Training in At-Risk CVD Groups

The model for identifying the predictors of intention to engage in physical exercise in the at-risk CVD groups was significant (χ^2^ = 60.36, *p* < 0.001), with 7% of the variance explained (Table 3). Intention to engage in physical exercise was 0.40-times lower in women than in men (OR: 0.40, 95% CI: 0.27–0.61) and 0.73-times lower in the group with high WHtR than in the group with low WHtR (Table 3).

The model for identifying the predictors of intention to engage in muscle training in the at-risk CVD groups was significant (χ^2^ = 22.62, *p* = 0.002), with 3.9% of the variance explained. Intention to engage in muscle training was 1.85-times higher in those with hypertension than in those who were not diagnosed with hypertension (OR: 1.85, 95% CI: 1.18–2.90) and 1.69-times higher among those with a BMI > 25 kg/m^2^ than in those with a lower BMI (OR: 1.69, 95% CI: 1.23–2.32) (Table 3).

## 4. Discussion

This study presents valuable foundational data for exploring fundamental strategies to lower the incidence of cardiovascular and cerebrovascular disease and develop intervention programs that improve compliance by identifying people’s willingness to modify their lifestyle according to their risk of cardiovascular and cerebrovascular disease. This study may provide the basis for planning and implementing health measures aimed at reducing modifiable risk factors for cardiovascular diseases [20].

In this study, intention to quit smoking was higher with increasing age in women than in men, with higher muscle training frequency, and with higher weekly drinking frequency in people at an intermediate or a high risk for cardiovascular and cerebrovascular disease (FRS ≥ 10%). These results are contradictory to previous findings on adults and older adults in public health centers in Korea, where men were more willing to quit smoking than women [21,22]. A previous review [23] of studies on cardiovascular risk assessment concluded that the predictive ability of a CVD risk score depends on the population being assessed. Therefore, the tools need to be epidemiologically relevant to the population. More research is needed to determine an appropriate and reliable tool.

The higher intention to quit smoking among women in our study seems to reflect the social trend that still renders female smoking taboo. Furthermore, people who frequently engaged in muscle training were more willing to quit smoking, suggesting that people who practice health behaviors are more likely to self-examine the effects of their health behaviors and modify their lifestyle [24]. Thus, specific information about the effects of quitting smoking and physical activity on health should be used to motivate people at risk of cardiovascular and cerebrovascular diseases to modify their lifestyle.

In this study, old age, elevated WHtR, and current smoking were identified as the predictors of intention to quit drinking. Similarly, a previous cohort study on adults aged 40–69 years showed that tendency to quit drinking increased with advancing age [25]. Furthermore, while smoking, drinking, and WHtR increased with increasing FRS, the high-risk group engaged in less physical activity, calling for strategies that promote specific lifestyle behaviors. These results are similar to those of a Lebanese study, where more than 70% of the participants chose the socially desirable answer but demonstrated low compliance with physical exercise, weight loss, and smoking cessation [26].

Increased WHtR indicates abdominal obesity and because the risk for CVD is higher in those with greater visceral fat even with the same body weight [27], management of abdominal obesity is highly important. Physical exercise is a crucial intervention to reduce abdominal obesity [28]. However, the 2014 KNHANES reports showed that compliance with moderate- or high-intensity physical activity in middle-aged adults (45–64 years) is only 22.9% in men and 16.4% in women [5], highlighting the urgency of developing physical exercise intervention programs that can reduce central obesity. The reasons for not increasing physical activity despite having CVD risk factors were identified as lack of knowledge about the benefits of exercise and lack of positive attitude toward exercise [29]. Hence, it is important to first motivate individuals to engage in physical activity and increase their self-efficacy [30]. Furthermore, women had 0.40-times lower intention to engage in physical exercise than men, and people with high WHtR were 0.73-times less willing to engage in physical exercise than those with low WHtR. These results are in line with previous findings on 1655 adults in Spain, where physical activity frequency decreased with increasing abdominal obesity and being overweight [28]. Although it is difficult to directly compare our results with the literature because of the lack of studies that directly asked about intention to exercise, a study comparing 2147 men and women in Taiwan reported that exercise compliance was higher in men than in women [31], which is in line with our findings. Therefore, subsequent studies should examine compliance with and willingness to engage in exercise between men and women and develop customized exercise programs for both sexes.

In the present study, hypertension and obesity were confirmed as the predictors of intention to engage in muscle training in at-risk CVD groups. As emphasized by the AHA guidelines [19], with the importance of muscle training in preventing CVD and in lowering the associated mortality, muscle training becomes more important with advancing age [32]. Our results showed that only 20.3% of the intermediate-risk group and 24.9% of the high-risk group engaged in muscle training, hence highlighting the importance of strategies that instill self-efficacy and inform individuals about the outcomes of exercise, for example it is recommended, to promote muscle training [33].

Additionally, in terms of lifestyle and comorbidities according to FRS in this study, the rates of smoking and drinking in the high-risk group were 75.6% and 41.6%, respectively, while the percentages of those engaging in high-intensity exercise and muscle training were 12.4% and 24.9%, respectively. This shows that people at high risk for CVD fail to modify their lifestyle even though they need to further strive to reduce their risk factors and manage their health [34].

This study has a few limitations. First, the sample was conveniently sampled, thus the findings have limited generalizability. Therefore, a multicenter study that utilizes tools to examine various cardiovascular and cerebrovascular disease risk factors should be conducted. Second, this was a cross-sectional study that investigated the intention to modify lifestyle using a self-report questionnaire, so respondent bias may exist. In addition, causality between cardiovascular disease and lifestyle cannot be determined. Therefore, longitudinal studies that implement a long-term lifestyle modification intervention and examine its effects on reducing the prevalence of cardiovascular and cerebrovascular disease are needed. Despite these limitations, this study is meaningful in that it confirmed the intention to modify the lifestyle habits for the risk factors of cardiovascular disease, centering on those with high cardiovascular risk who actually need lifestyle improvement.

## 5. Conclusions

This study identified the predictors of intention to modify lifestyle behaviors like smoking, drinking, physical activity, and muscle training, in people at risk for CVD. The findings were based on the FRS data of 1229 individuals aged 30–74 years without cardiovascular and cerebrovascular disease who visited a health examination center in Korea.

The results showed that old age, being female, high muscle training frequency, and high drinking frequency are predictors of intention to quit smoking, while old age, low WHtR, and current smoking are the predictors of intention to quit drinking in at-risk CVD groups. Further, intention to engage in physical activity was 0.27-times lower among women; that is, intention to engage in physical activity was higher for males and those with low WHtR (no abdominal obesity), whereas the intention to engage in muscle training was higher in those with hypertension and higher BMI.

Intention needs to be supplemented by other, more proximal factors that might compromise or facilitate the enactment of intentions. These differences can be overcome by promoting self-efficacy for behavior modification and establishing educational strategies for specific behavior modification, which may help bridge the intention–behavior gap.

## Figures and Tables

**Table 1:** Differences in general characteristics and intention to modify lifestyle according to FRS (*N* = 1229).

Variable	Category	Low-Risk Group (*n* = 774)	Intermediate-Risk Group (*n* = 246)	High-Risk Group (*n* = 209)	χ^2^	*p*
		*n* (%) or M±SD				
Age (years) *		54.6 ± 10.7	62.0 ± 7.7	65.4 ± 5.7	135.19	<0.001
Sex	Male	97 (12.5)	97 (39.4)	184(88.0)	451.40	<0.001
	Female	677 (87.5)	149 (60.6)	25(12.0)		
DM	Yes	34 (4.4)	42 (20.6)	47(22.5)	76.87	<0.001
	No	740 (95.6)	204 (79.4)	162 (77.5)		
Hypertension	Yes No	97 (12.5) 677 (87.5)	77 (31.3) 169 (68.7)	96 (45.9) 113 (54.1)	122.72	<0.001
Dyslipidemia	Yes No	68 (8.8) 706 (91.2)	34 (13.8) 212 (86.2)	14 (6.7) 195 (93.3)	7.75	0.21
Current smoking	Yes No	77 (10.0) 696 (90.0)	74 (30.1) 172 (69.9)	158 (75.6) 51 (24.4)	380.34	<0.001
Alcohol consumption (more than once/week)	Yes	152 (19.6)	56 (22.8)	87 (41.6)	43.88	<0.001
	No	622 (80.4)	190 (77.2)	122 (58.4)		
Vigorous physical activity (over 150 min/week)	Yes	116 (15.0)	32 (13.0)	26 (12.4)	1.21	0.543
	No	658 (85.0)	214 (87.0)	183 (87.6)		
Moderate physical activity (over 300 min/week)	Yes	143 (18.5)	55 (22.4)	50 (23.9)	3.94	0.140
	No	631 (81.5)	191 (77.6)	159 (76.1)		
Muscle strengthening exercise (more than twice/week)	Yes	146 (18.9)	50 (20.3)	52 (24.9)	3.70	0.157
	No	628 (81.1)	196 (79.7)	157 (75.1)		
Intention to quit smoking	Yes	46 (5.9)	30 (12.2)	49 (23.4)	56.55	<0.001
	No	728 (94.1)	216 (87.8)	160 (76.6)		
Intention to stop drinking	Yes	148 (19.1)	47 (19.1)	59 (28.2)	8.78	0.012
	No	626 (80.9)	199 (80.9)	150 (71.8)		
Intention to dophysical activity	Yes	293 (37.9)	89 (36.2)	85 (40.7)	0.98	0.611
	No	481 (62.1)	157 (63.8)	124 (59.3)		
Intention to do strength exercise	Yes	604 (78.0)	192 (78.0)	151 (72.2)	3.29	0.193
	No	170 (22.0)	54 (22.0)	58 (27.8)		

Abbreviations: SD: standard deviation; DM: diabetes mellitus; FRS: Framingham risk score; Framingham risk score classification: high-risk group (FRS ≥ 20%), intermediate-risk group (FRS: 10–19%), Low-risk group (FRS < 10%). * analyzed by ANOVA.

**Table 2:** Differences in hematologic tests according to FRS (*N* = 1229).

Variable		Low Risk Group (*n* = 774)	Intermediate Risk Group (*n* = 246)	High Risk Group (*n* = 209)	F or χ^2^	*p*
		n (%) or M ± SD				
BP (mmHg)	Systolic	117.2 ± 10.1	126.9 ± 12.2	127.4 ± 14.1	110.84	<0.001
	Diastolic	72.7 ± 7.9	76.7 ± 8.9	76.6 ± 9.3	31.88	<0.001
BMI (kg/m^2^)		23.8 ± 3.1	24.9 ± 3.1	24.9 ± 2.9	16.61	<0.001
WHtR		0.51 ± 0.1	0.53 ± 0.1	0.53 ± 0.1	15.94	<0.001
Fasting glucose (mg/dL)		95.0 ±15.9	204 ± 82.9	110.1 ± 31.7	47.19	<0.001
HDL cholesterol(mg/dL)		57.5 ± 11.4	51.0 ± 10.4	47.5 ± 9.8	84.96	<0.001
Total cholesterol (mg/dL)		202.1 ± 37.9	201.9 ± 44.0	191.5 ± 45.2	5.90	0.003
AST (mg/dL)		25.8 ± 10.3	27.0 ± 9.0	30.4 ± 23.4	10.19	<0.001
GGT (IU/L)		26.0 ± 43.2	32.2 ± 30.6	52.6 ± 112.8	16.53	<0.001
Cr (mg/dL)		0.8 ± 0.1	0.9 ± 0.15	1.0 ± 0.1	149.88	<0.001

Abbreviations: SD: standard deviation; BP: blood pressure; BMI: body mass index; WHtR: waist to height ratio; HDL: high-density lipoprotein; AST: aspartate aminotransferase; GGT: gamma glutamyl transpeptidase; Cr: Creatinine.

**Table 3:** Predictors of intention to modify lifestyle in at-risk CVD groups (N = 455).

Outcome Variable	B	*p*	OR	95% CI	
Factor				Lower	Upper
Intention to quit smoking					
**Age**	**0.14**	**<0.001**	**1.15**	**1.11**	**1.20**
**Sex** **^†^**	**1.78**	**<0.001**	**5.95**	**3.18**	**11.13**
**Frequency of strength exercise (per week)**	**0.16**	**0.015**	**1.17**	**1.03**	**1.33**
**Frequency of drinking (per week)**	**0.24**	**0.009**	**1.27**	**1.06**	**1.51**
WHtR ^†^	0.37	0.129	1.45	0.90	2.33
Model test: Chi-square = 294.09, *p* < 0.001, Nagelkerke *R*^2^ = 0.418					
Intention to stop drinking					
**Age**	**0.73**	**<0.001**	**1.08**	**1.05**	**1.10**
Sex ^†^	0.36	0.117	1.44	0.91	2.27
Frequency of strength exercise per week)	−0.02	0.650	0.10	0.90	1.07
Smoking ^†^	1.33	<0.001	3.78	2.39	6.00
WHtR ^†^	−**0.39**	**0.028**	**0.68**	**0.48**	**0.96**
Model test: Chi-square = 15.84, *p* < 0.001, Nagelkerke *R^2^* = 0.243					
Intention to do physical exercise					
Age	−0.02	0.201	0.99	0.97	1.00
**Sex** **^†^**	−**0.91**	**<0.001**	**0.40**	**0.27**	**0.61**
Smoking ^†^	0.03	0.909	1.03	0.67	1.59
Frequency of drinking (per week)	0.03	0.990	1.00	0.65	1.54
WHtR ^†^	−**0.31**	**0.041**	**0.73**	**0.54**	**0.99**
Model test: Chi-square = 60.36, *p* < 0.001, Nagelkerke *R^2^*= 0.070					
Intention to do strength exercise					
Age	0.01	0.160	1.01	1.00	1.03
Sex ^†^	0.40	0.161	1.50	0.85	2.64
Smoking ^†^	0.05	0.881	1.05	0.58	1.88
Frequency of drinking (per week)	−0.32	0.115	0.73	0.49	1.08
Diabetes mellitus	0.33	0.359	1.40	0.68	2.89
**Hypertension** **^†^**	**0.62**	**0.007**	**1.85**	**1.18**	**2.90**
**Body mass index** **^†^**	**0.52**	**0.001**	**1.69**	**1.23**	**2.32**

Abbreviations: CI confidence interval; OR: odds ratio; FRS: Framingham risk score. † Reference: Sex: Male; WHtR: ≤0.5; Smoking: No; Body Mass Index: ≤25; Hypertension: No; Diabetes mellitus: No; Bold indicates statistical significance at *p* < 0.05.

## Data Availability

The data presented in this study are available on request from the first author.
